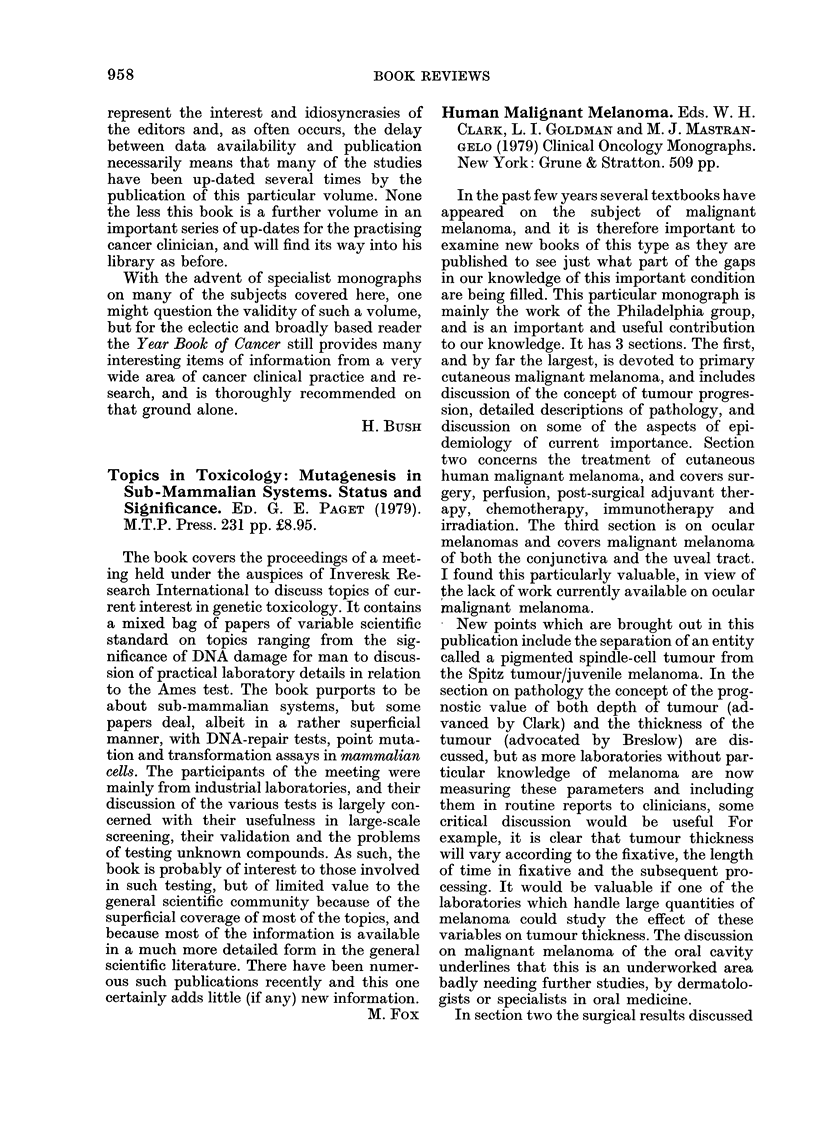# Topics in Toxicology: Mutagenesis in Sub-Mammalian Systems. Status and Significance

**Published:** 1979-12

**Authors:** M. Fox


**Topics in Toxicology: Mutagenesis in Sub-Mammalian Systems. Status and Significance.** Ed. G. E. Paget (1979). M.T.P. Press. 231 pp. £8.95.

The book covers the proceedings of a meeting held under the auspices of Inveresk Research International to discuss topics of current interest in genetic toxicology. It contains a mixed bag of papers of variable scientific standard on topics ranging from the significance of DNA damage for man to discussion of practical laboratory details in relation to the Ames test. The book purports to be about sub-mammalian systems, but some papers deal, albeit in a rather superficial manner, with DNA-repair tests, point mutation and transformation assays in *mammalian cells*. The participants of the meeting were mainly from industrial laboratories, and their discussion of the various tests is largely concerned with their usefulness in large-scale screening, their validation and the problems of testing unknown compounds. As such, the book is probably of interest to those involved in such testing, but of limited value to the general scientific community because of the superficial coverage of most of the topics, and because most of the information is available in a much more detailed form in the general scientific literature. There have been numerous such publications recently and this one certainly adds little (if any) new information.

M. Fox